# Intradermal Injection of a Protein Alone Without Additional Adjuvants Using a Needle-Free Pyro-Drive Jet Injector Induces Potent CD8^+^ T Cell-Mediated Antitumor Immunity

**DOI:** 10.3390/ijms26094442

**Published:** 2025-05-07

**Authors:** Jukito Sonoda, Izuru Mizoguchi, Natsuki Yamaguchi, Eri Horio, Satomi Miyakawa, Mingli Xu, Toshihiko Yoneto, Yasuhiro Katahira, Hideaki Hasegawa, Takashi Hasegawa, Kunihiko Yamashita, Takayuki Yoshimoto

**Affiliations:** 1 Department of Immunoregulation, Institute of Medical Science, Tokyo Medical University, 6-1-1 Shinjuku, Shinjuku-ku, Tokyo 160-8402, Japan; 2 Department of Device Application for Molecular Therapeutics, Graduate School of Medicine, Osaka University, CoMIT 0603, 2-2 Yamada-oka, Suita 565-0871, Osaka, Japanku_yamashita@jp.daicel.com (K.Y.); 3 Medical Device Division, Life Sciences Strategic Business Unit, Daicel Corporation, 2-2 Yamada-oka, Suita 565-0871, Osaka, Japan

**Keywords:** cancer vaccine, generation of CTL, physical adjuvant, pyro-drive jet injector, shear stress

## Abstract

Vaccines usually contain an adjuvant that activates innate immunity to promote the acquisition of adaptive immunity. Aluminum and lipid nanoparticles have been used for this purpose, but their accumulation or widespread circulation in the body can lead to adverse effects. In contrast, physical adjuvants, which use physical energy to transiently stress tissues, do not persist in exposed tissues or cause lasting adverse effects. Herein, we investigate the effects of intradermal injection of endotoxin-free ovalbumin (OVA) protein alone without additional adjuvants using a needle-free pyro-drive jet injector (PJI) on tumor vaccination efficacy. Intradermal injection of OVA protein alone using PJI significantly increased OVA-specific CD8^+^ T cell expansion in the lymph node, although lymph node swelling was much less than when aluminum hydroxide was used. The injection also induced OVA-specific killing activity and antibody production and showed strong CD8^+^ T cell-dependent prophylactic antitumor effects against transplanted E.G7-OVA tumors. In particular, intradermal injection of the fluorescent OVA protein significantly enhanced its uptake by XCR1^+^ dendritic cells, which have a strong ability to cross-present extracellular proteins in the skin and draining lymph nodes. In addition, the injection increased the expression of HMGB1, one of the potent danger signals whose expression has been reported to increase in response to shear stress. Thus, intradermal injection of OVA protein alone without any additional adjuvants using PJI induces potent CD8^+^ T cell-mediated antitumor immunity by enhancing its uptake into XCR1^+^ dendritic cells, which have a high cross-presentation capacity accompanied by an increased expression of shear stress-induced HMGB1.

## 1. Introduction

Messenger RNA vaccines against severe acute respiratory syndrome coronavirus type 2 (SARS-CoV-2), which are currently being used successfully around the world [1,2], have shown strong prophylactic efficacy and safety when injected intramuscularly with a needle syringe [3]. Vaccines typically contain a target antigen protein and an adjuvant that activates innate immunity to promote the acquisition of adaptive immunity [4]. Although COVID-19 mRNA vaccines are currently saving many lives, adverse effects such as cytokine release syndrome and acute myocardial infarction have been reported [5,6,7]. As a mechanism for these side effects, it has been suggested that COVID-19 mRNA is encapsulated in lipid nanoparticles (LNPs) [8], which are widely distributed in the human body after intramuscular injection and may exert pro-inflammatory effects [9]. In addition, COVID-19 mRNA encodes a transmembrane SARS-CoV-2 spike protein whose antigens can be shed into the circulation and whose binding to angiotensin-converting enzyme 2 can lead to vaccine-related adverse effects [10,11].

In general, adjuvants are danger signals such as pathogen-associated molecular patterns (PAMPs) and damage-associated molecular patterns (DAMPs) [12,13]. To date, only a limited number of adjuvants have been approved for use in humans due to toxicity. Aluminum adjuvants have been approved for human use by the US FDA and are used worldwide [12,14,15]. Under normal physiological conditions, aluminum is excreted by the kidneys, but under conditions of renal impairment, aluminum accumulation in the body can lead to severe toxicity, causing effects such as neurological symptoms [16]. Due to safety concerns about such chemical adjuvants, safer and more effective methods are now required. Physical adjuvants, which use physical energy to stress tissues, do not use chemical or biological substances and therefore do not persist in exposed tissues or cause lasting adverse effects [17,18,19]. The skin is an ideal site for vaccination because it contains large numbers of immune cells, including dendritic cells (DCs), which are professional antigen-presenting cells [20,21]. However, intradermal injections require more technical skill than intramuscular injections and are less suitable for mass vaccination. In addition, needle syringe injections are painful and can lead to needle phobia and needlestick injuries [22]. To overcome these problems, needle-free delivery devices such as electroporation, thermal ablation, lasers, and microneedles have been developed to enhance cellular and humoral immunity painlessly and efficiently by intradermal injection [20,23,24,25].

Recently, a novel needle-free pyro-drive jet injector (PJI) called Actranza^TM^ lab (Daicel Corporation, Osaka, Japan) has been developed, which is unique in that the PJI uses gunpowder as the mechanical driving force, especially bi-phasic pyrotechnics, to induce high jet velocity and wide dispersion of the injected plasmid DNA solution in the skin [26]. Luciferase- or ovalbumin (OVA)-expressing DNA was efficiently delivered to dermal tissue by intradermal injection using the PJI, and protein expression was found to be much higher than using a typical needle syringe [27,28]. This effect is most likely due to the increased uptake of the plasmid by the shear stress generated by the high jet velocity of the PJI [29]. In addition, the production of OVA-specific antibodies was greatly enhanced [28]. Similarly, intradermal injection of DNA expressing the SARS-CoV-2 spike protein using the PJI resulted in increased production of neutralizing antibodies against the virus and inhibition of viral infection in a mouse model [30]. Recently, we demonstrated that intradermal injection of OVA-expressing plasmid DNA into mice using a PJI greatly enhanced OVA-specific CD8^+^ T cell-mediated potent antitumor effects against transplanted E.G7-OVA tumors [31].

During the course of these studies, we serendipitously discovered that intradermal injection of OVA protein alone, without additional adjuvants, instead of its expressing plasmid can induce potent OVA-specific CD8+ T cell expansion in the draining lymph node. When the DNA plasmid was used, it was difficult to conclude this because DNA itself is one of the danger signals. This preliminary result led us to believe that intradermal injection using the PJI itself may indeed induce activation of innate immune responses via the generation of shear stress as a physical adjuvant. Therefore, in the present study, we sought to clarify this possibility and its possible underlying mechanisms. As a model protein antigen, we used endotoxin-free OVA protein, because commercially available OVA protein is known to be contaminated with endotoxin [32]. Similar to plasmid DNA, intradermal injection of OVA protein using the PJI greatly enhanced OVA-specific CD8^+^ T cell-mediated strong prophylactic antitumor effects against transplanted E.G7-OVA tumors. Notably, intradermal injection greatly enhanced the uptake of OVA protein by XCR1^+^MHC class II^+^CD11c^+^ DCs, which have a strong ability to cross-present extracellular proteins [33]. In addition, PJI injection increased the skin expression of high mobility group box 1 (HMGB1), one of the potent danger signals whose expression has been reported to increase in response to shear stress [34,35]. This is the first report showing that vaccination with a protein alone, without additional adjuvants, using the physical adjuvant PJI induces the antigen-specific CD8^+^ T cell-mediated antitumor immunity.

## 2. Results

### 2.1. Intradermal Injection of OVA Protein Using PJI Efficiently Increases OVA-Specific CD8^+^ T Cell Generation with Less Lymph Node Swelling

**Figure 1:**
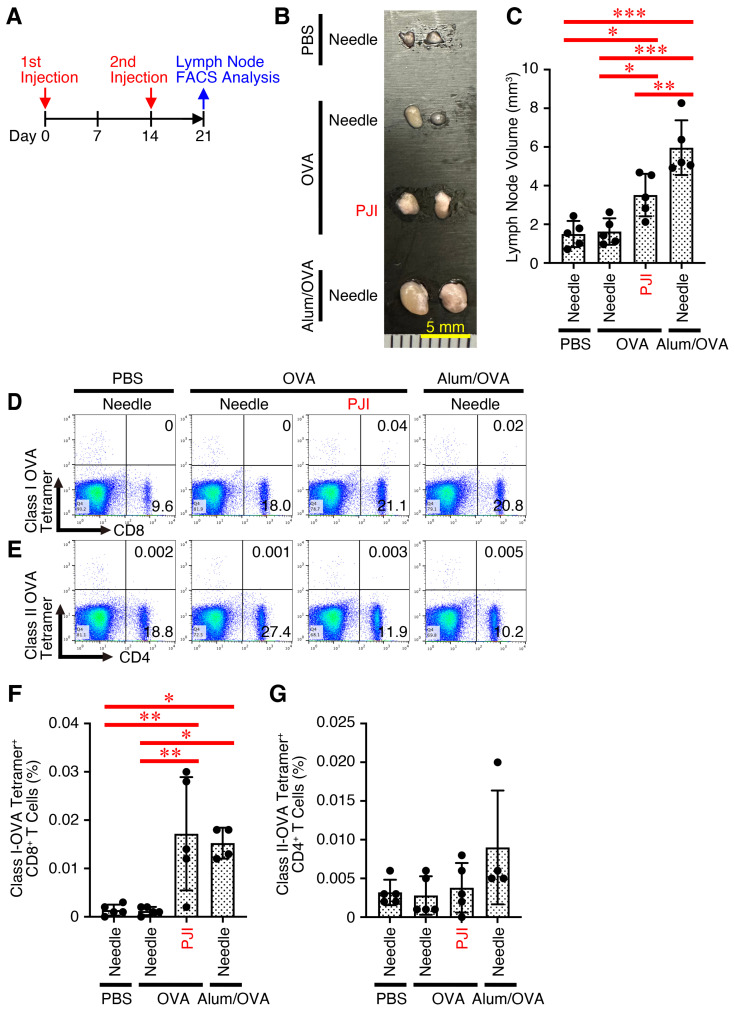
Intradermal injection of OVA protein using PJI efficiently increases OVA-specific CD8^+^ T cell generation with less lymph node swelling. Mice were injected intradermally with OVA protein using PJI or PBS, OVA protein alone and OVA protein combined with Alum (Alum/OVA) using a needle syringe twice with a 2-week interval (**A**). One week later, the draining lymph nodes were removed and their appearance was photographed. Representative photographs are shown (**B**,**C**) and average volumes were calculated and compared (**D**,**E**). Lymph node cells were then stained with OVA-specific MHC class I tetramer and anti-CD8, or OVA-specific MHC class II tetramer and anti-CD4. Representative dot plots are shown (**D**,**E**), and average percentages of positive cells were calculated and compared (**F**,**G**). Data shown are the mean ± SD (*n* = 4~5) of three independent experiments. *p* values were determined by one-way ANOVA with the Tukey’s test for multiple comparisons. * *p* < 0.05, ** *p* < 0.01, *** *p* < 0.001.

First, we investigated whether intradermal injection of an endotoxin-free protein, OVA, as a model antigen using PJI was sufficient to induce immune responses against the protein without additional adjuvants. To investigate this possibility, mice were injected intradermally with OVA protein using PJI or PBS, OVA protein alone and OVA protein combined with Alum (Alum/OVA) twice with a 2-week interval using a needle syringe (Figure 1A). One week after the second injection, the draining inguinal lymph nodes were removed, and their appearance and size were compared among them (Figure 1B,C). Injection of Alum/OVA with a needle syringe increased lymph node size much more than OVA protein with a PJI. There was no difference in the size between PBS and OVA protein using a needle syringe. Notably, however, analysis using the tetramer staining assay showed that PJI increased the frequency of OVA-specific CD8^+^ T cells at a level similar to that of Alum (Figure 1D,F). In contrast, the frequency of OVA-specific CD4^+^ T cells was not significantly increased (Figure 1E,G).

Taken together, these results suggest that intradermal injection of OVA protein alone using the PJI, even without additional adjuvants, significantly increases the generation of antigen-specific CD8^+^ T cells but not CD4^+^ T cells, to the level induced by Alum with much less lymph node swelling compared to Alum.

### 2.2. Intradermal Injection of OVA Protein Using PJI Efficiently Increases Killing Activity

**Figure 2:**
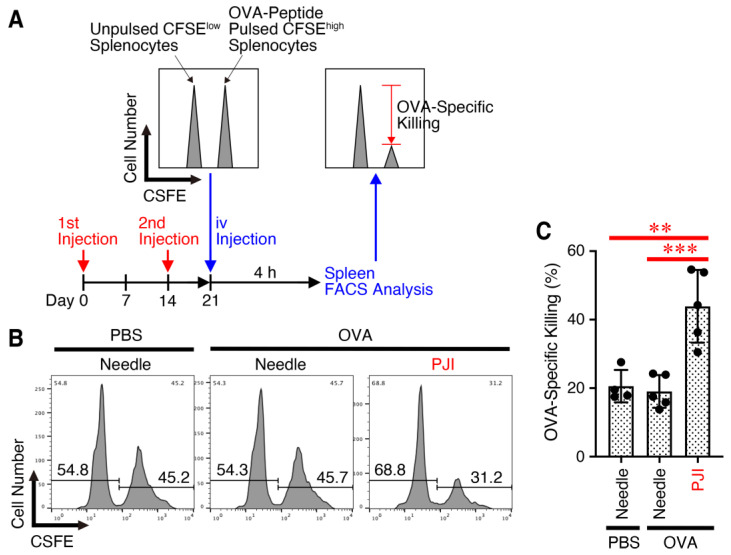
Intradermal injection of OVA protein using PJI efficiently increases killing activity. Mice were injected intradermally with OVA protein using PJI or PBS and OVA protein using a needle syringe twice with a 2-week interval (**A**). One week later, mice received equal numbers of MHC class I-restricted OVA_257–264_ peptide-pulsed CFSE^high^ cells and unpulsed CFSE^low^ cells, and OVA-specific in vivo killing activity in the splenocytes was analyzed by flow cytometry. Representative histograms are shown in (**B**), and the average specific killing was calculated and compared in (**C**). Data shown are the mean ± SD (*n* = 4~5) of two independent experiments. *p* values were determined by one-way ANOVA with the Tukey’s test for multiple comparisons. ** *p* < 0.01, *** *p* < 0.001.

We then investigated whether the generation of OVA-specific CD8^+^ T cells would lead to an increase in antigen-specific killing activity. For the in vivo killing assay, mice were injected intradermally with OVA protein using PJI or a needle syringe or with PBS using a needle syringe twice with a 2-week interval (Figure 2A). One week later, the mice received an equal number of MHC class I-restricted OVA_257–264_ peptide-pulsed CFSE^high^ splenocytes and unpulsed CFSE^low^ splenocytes. OVA-specific in vivo killing activity in the spleen was then analyzed by flow cytometry. Injection with the PJI, but not with a needle syringe, significantly increased antigen-specific in vivo killing activity (Figure 2B,C).

Thus, these results suggest that intradermal injection of OVA protein using PJI, even without additional adjuvants, significantly enhances the functional activity of CD8^+^ T cells, including antigen-specific killing activity.

### 2.3. Intradermal Injection of OVA Protein Using PJI Efficiently Enhances Antibody Production

**Figure 3:**
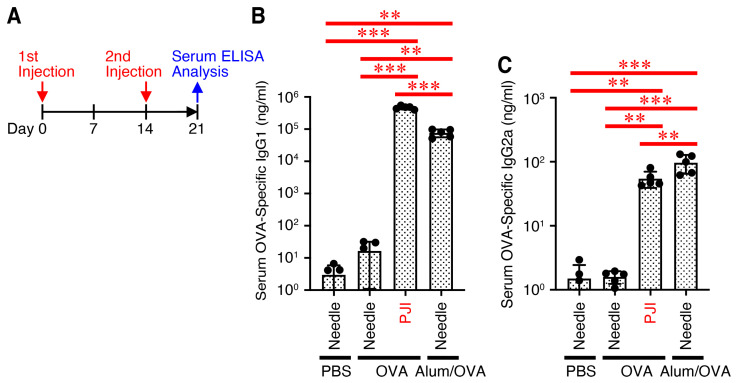
Intradermal injection of OVA protein using PJI efficiently enhances antibody production. Mice were injected intradermally with OVA protein using PJI or PBS, OVA protein alone, and OVA protein combined with Alum (Alum/OVA) twice with a 2-week interval using a needle syringe (**A**). One week later, the titers of anti-OVA IgG1 (**B**) and IgG2a (**C**) isotype antibodies in serum were measured. Data shown are the mean ± SD (*n* = 5) of three independent experiments. *p* values were determined by one-way ANOVA with the Tukey’s test for multiple comparisons. ** *p* < 0.01, *** *p* < 0.001.

Since the generation of antigen-specific CD4^+^ T cells was not pronounced by injection using PJI (Figure 1E,G) but could be induced, we next investigated whether an injection of OVA protein increased antibody production. Mice were injected intradermally with OVA protein using the PJI or PBS, OVA protein, and Alum/OVA using a needle syringe twice at 2-week intervals (Figure 3A). One week later, serum OVA-specific antibody titers were determined. Intradermal injection of OVA protein using a PJI but not a needle syringe significantly increased both of the antibody titers of OVA-specific IgG1 and IgG2a isotypes to levels induced by Alum/OVA (Figure 3B,C).

Thus, intradermal injection of OVA protein using PJI enhances not only the CD8^+^ T cell response but also the CD4^+^ T cell response, including the specific antibody production through potential Th1 and Th2 immune responses.

### 2.4. Intradermal Injection of OVA Protein Using the PJI Shows Potent CD8^+^ T Cell-Dependent Prophylactic Antitumor Effects Against the E.G7-OVA Tumors

**Figure 4:**
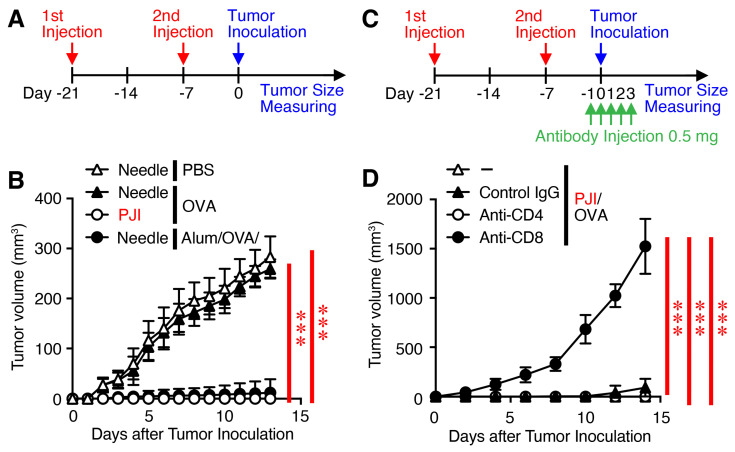
Intradermal injection of OVA protein using PJI shows potent CD8^+^ T cell-dependent prophylactic antitumor effects against the E.G7-OVA tumors. Mice were injected intradermally with OVA protein using PJI or PBS, OVA protein alone, and OVA protein combined with Alum using a needle syringe twice with a 2-week interval (**A**). One week later, mice were subcutaneously inoculated with E.G7-OVA tumors, and tumor growth was monitored (**B**). For depletion of CD4^+^ T cells or CD8^+^ T cells, each mouse was injected i.p. with 0.5 mg rat anti-mouse CD8, anti-CD4, or normal rat IgG as control antibody, in 200 μL PBS, one day before tumor inoculation and once daily for the following 4 consecutive days (**C**), and tumor growth was monitored (**D**). In (**D**), there are triangles just behind the circles. Data shown are the mean ± SD (*n* = 5) of two independent experiments. *p* values were determined by two-way ANOVA with the Tukey’s test for multiple comparisons. *** *p* < 0.001.

Since thus the injection of OVA protein using the PJI induced both CD8^+^ T cell and possibly CD4^+^ T cell-mediated immune responses, we next investigated whether the injection of OVA protein induced an antigen-specific vaccination effect against the progression of transplanted E.G7-OVA tumors. Mice were intradermally injected with OVA protein using PJI or PBS, OVA protein, and Alum/OVA using a needle syringe twice with a 2-week interval (Figure 4A). One week later, mice were inoculated subcutaneously with E.G7-OVA tumors, and tumor growth was monitored. Tumor growth was almost completely inhibited in mice injected with OVA protein by PJI and with Alum/OVA by a needle syringe (Figure 4B). In contrast, a similar inhibition of tumor growth was not observed in mice injected with PBS or OVA protein using a needle syringe. To further investigate which immune responses are critical for the induction of protective immunity, depletion experiments were performed using anti-CD4, anti-CD8, or control IgG (Figure 4C). Depletion of CD8^+^ T cells, but not CD4^+^ T cells, completely reversed the prophylactic effect (Figure 4D).

These results suggest that intradermal injection of OVA protein using PJI induces prophylactic antitumor effects in a mainly CD8^+^ T cell-dependent manner.

### 2.5. Intradermal Injection of OVA Protein Using PJI Greatly Enhances Its Uptake into XCR1^+^DCs with High Cross-Presentation Ability

**Figure 5:**
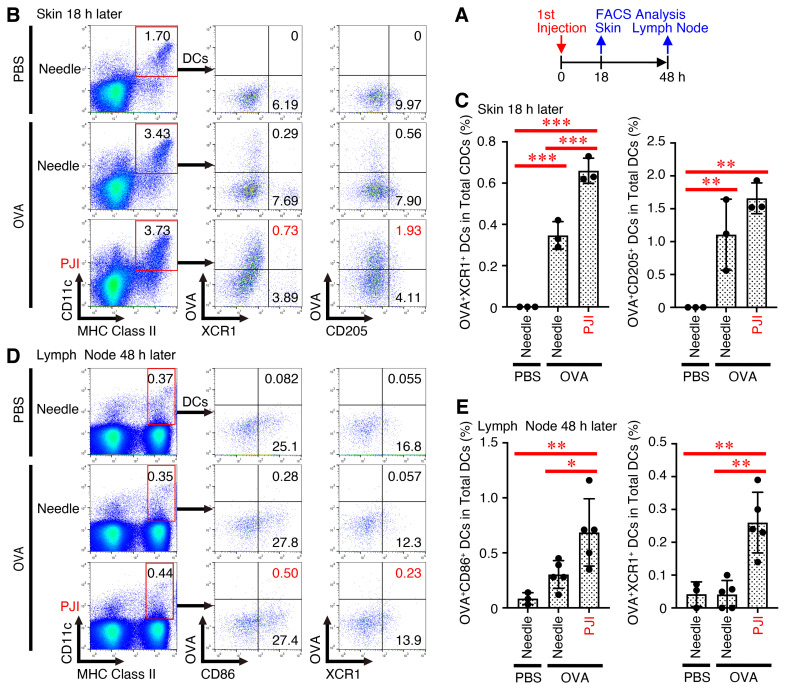
Intradermal injection of OVA protein using PJI greatly enhances its incorporation into XCR1^+^DCs with high cross-presentation ability. Mice were injected intradermally with OVA protein conjugated with Alexa Flour 647 using PJI or a needle syringe and PBS using a needle syringe (**A**). Eighteen hours later, the skin section was excised and digested with collagenase, and the resulting cells were stained with anti-CD11c, anti-MHC class II, anti-XCR1, and anti-CD205 antibodies, followed by FACS analysis (**B**,**C**). Twenty-four hours later, lymph node cells were similarly stained and subjected to FACS analysis (**D**,**E**). Representative dot plots are shown (**B**,**D**), and the average cell population was calculated and compared (**C**,**E**). Data shown are the mean ± SD (*n* = 3~5) of two independent experiments. *p* Values were determined by one-way ANOVA with the Tukey’s test for multiple comparisons. * *p* < 0.05, ** *p* < 0.01, *** *p* < 0.001.

To address the question of why intradermal injection of OVA protein using PJI preferentially activated CD8^+^ T cells, we next investigated the involvement of cross-presentation. In general, endogenous proteins are processed via the MHC class I pathway for presentation to CD8^+^ T cells, whereas exogenous proteins are processed via the MHC class II pathway for presentation to CD4^+^ T cells [36]. However, specialized antigen-presenting cells such as DCs can present exogenous proteins on MHC class I to CD8^+^ T cells, a process called cross-presentation [37]. To date, several cell surface markers have been identified for cross-presenting DCs. Of these markers, the most commonly found molecule is the chemokine receptor XCR1 [33,38,39]. Therefore, we next used Alexa Flour 647-conjugated OVA protein to determine which cell populations take up the OVA protein most efficiently. Mice were injected intradermally with the fluorescence-labeled OVA protein using a PJI or needle syringe and with PBS using a needle syringe. Eighteen hours later, the skin section was excised and digested with collagenase, and the resulting cells were analyzed by flow cytometer (Figure 5A). Forty-eight hours later, the draining lymph node cells were analyzed in a similar manner. In the skin section, the percentage of OVA^+^XCR1^+^ DCs among the total CD11c^+^MHC class II^+^ DCs induced by PJI injection was much higher than that induced by needle injection (Figure 5B,C). The percentage of another marker for cross-presenting DCs, CD205 [40], showed a similar trend. In the draining lymph node cells, the percentage of mature OVA^+^CD86^+^ DCs and also OVA^+^XCR1^+^ cells in the total DCs induced by PJI was much higher than that induced by needle injection (Figure 5D,E).

These results suggest that the potent activation of CD8^+^ T cells and the resulting CD8^+^ T cell-dependent antitumor immune responses are likely due to the preferential uptake of OVA protein by cross-presenting DCs.

### 2.6. Intradermal Injection of OVA Protein Using PJI Increases Protein Expression of HMGB1 in the Skin

**Figure 6:**
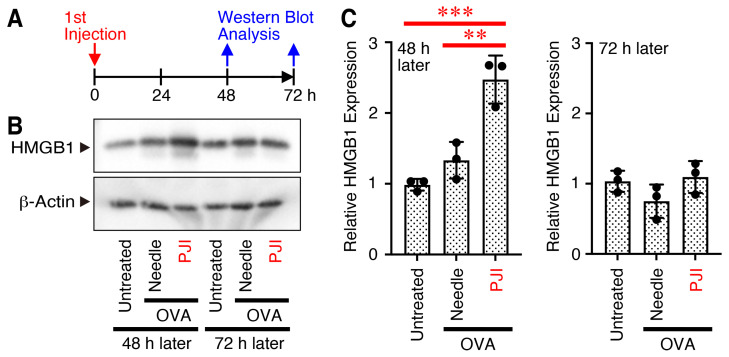
Intradermal injection of OVA protein using PJI increases protein expression of HMGB1 in the skin. Mice were injected intradermally with OVA protein using PJI or a needle syringe. 48 h and 72 h later, the skin section was excised and lyzed in a RIPA buffer (**A**). The resulting cell lysate was subjected to SDS-PAGE followed by western blot analysis using anti-HMGB1 and anti-actin (**B**). Full original images of the western blots are shown in Supplementary Figure S1. The intensities of the respective bands corresponding to HMGB1 and actin were measured using FIJI and compared (**C**). Data shown are the mean ± SD (*n* = 3) of two independent experiments. *p* Values were determined by one-way ANOVA with the Tukey’s test for multiple comparisons. ** *p* < 0.01, *** *p* < 0.001.

PJI has previously been shown to induce local shear stress due to the high jet velocity and to enhance plasmid DNA uptake and endocytosis, resulting in increased protein expression [29]. Of note, shear stress has also been reported to induce adenosine triphosphate (ATP) release [41], reactive oxygen species generation [42], and HMGB1 translocation [34], which led us to consider the activation of innate immunity, including DC maturation, by PJI-induced shear stress. Therefore, we next focused on one of the danger signals, HMGB1, and examined its expression after injection using PJI. Mice were injected intradermally with OVA protein using PJI or a needle syringe, and the skin section was then excised at 48 and 72 h and analyzed by western blot using the antibody against HMGB1 (Figure 6A). Intradermal injection using a PJI, but not a needle syringe, significantly increased protein expression of HMGB1 in the skin 48 h after injection and then decreased to basal levels (Figure 6B,C).

Thus, intradermal injection of OVA protein using PJI increased the protein expression of HMGB1 in the skin, which may contribute to the activation of innate immunity.

## 3. Discussion

In the present study, intradermal injection of OVA protein using PJI significantly increased OVA-specific CD8^+^ T cell expansion, induced OVA-specific killing activity and antibody production, and showed potent CD8^+^ T cell-dependent prophylactic antitumor effects against transplanted E.G7-OVA tumors. In particular, the intradermal injection of OVA protein significantly increased its uptake and endocytosis by XCR1^+^DCs with a strong cross-presenting ability. In addition, the injection increased the expression of HMGB1, one of the potent danger signals whose expression has been reported to be increased by shear stress [34,35]. Thus, intradermal injection of OVA protein alone without any additional adjuvants using PJI induces potent CD8^+^ T cell-mediated antitumor immunity, possibly with upregulation of its shear stress-induced HMGB1.

Tumor antigen-specific CD8^+^ T cell responses can be induced by cross-presentation of tumor antigens in DCs [43]. Efficient cross-presentation requires tumor cell death or cell damage and is associated with translocation or release of DAMPs [44]. Immunogenic cell death is generally accompanied by the surface translocation of “eat me” signals, such as calreticulin, from the endoplasmic reticulum, and secretion of DAMPs, such as ATP, heat shock protein 70 (HSP70), HMGB1, interleukin (IL)-1α, IL-33, and calreticulin from dying cells into the extracellular space [45]. HMGB1 is a non-histone chromatin-binding nuclear protein that plays an important role in chromatin structure and gene expression [46,47]. HMGB1 can also be released either by activated immune cells or passively released from damaged cells, and it is a potent endogenous alarm for innate immunity. There are several important receptors for HMGB1, including a receptor for advanced glycation end products (RAGE) and toll-like receptors (TLR)2 and TLR4 [48]. HMGB1 plays an important role in inflammation-associated diseases, including autoimmune diseases such as systemic lupus erythematosus and diabetes and malignant tumors by promoting the expression of pro-inflammatory cytokines. HMGB1 promotes the maturation and migration of DCs, thereby promoting T cell proliferation and differentiation of the Th1 response through binding to its receptor RAGE. Interestingly, HMGB1 released from dead cells can also act as an immune adjuvant by enhancing antigen immunogenicity, suggesting that HMGB1 could serve as an adjuvant for vaccine strategy [49,50]. During protein processing in DCs after endocytosis, the fusion between phagosomes and lysosomes, which leads to degradation of the dying cell rather than antigen presentation by DCs, is prevented by TLR4 activation by HMGB1, allowing optimal antigen presentation [51]. Thus, TLR4 and HMGB1 play a critical role in the cross-presentation of tumor antigens from dying tumor cells by DCs to CD8^+^ T cells [51]. These reports are consistent with the present findings that PJI-mediated upregulation of HMGB1 in the skin plays a critical role in the activation of innate immune responses, including DC maturation and cross-presentation, and consequent activation of CD8^+^ T cell-mediated immune responses.

Shear stress is a frictional force applied tangential or parallel to the surface of cells flowing in the blood, and it physiologically regulates the homeostasis of thrombosis and fibrosis via its critical sensors, endothelial cells [52]. Lower shear stress accelerates thrombosis and consequently atherosclerosis, whereas higher shear stress prevents thrombosis. In particular, shear stress has been reported to induce HMGB1 translocation [34], ATP release [41], and reactive oxygen species generation [42] in endothelial cells. Low shear stress in human umbilical vein endothelial cells has been reported to induce the translocation of HMGB1 from the nucleus to the cytoplasm and release to the supernatant, resulting in an inflammatory response [34]. Knockdown of HMGB1 by siRNA reduced shear stress-induced inflammatory responses such as intracellular adhesion molecule 1 expression, tumor necrosis factor-α and IL-1β secretion, and monocyte adhesion. Furthermore, using a novel microfluidic channel fabricated by soft lithography to mimic inflammatory edema, shear stress was shown to regulate the migration of DCs and upregulate their activation markers MHC class I and CD86 compared to DCs under static conditions [53].

The rapid local injection of a liquid under high pressure induces the uptake of molecules by cells by facilitating the internalization of plasma membrane proteins and enhancing endocytosis [54,55]. The PJI is a unique device that uses two different types of explosives, namely ignition and smokeless powders, allowing the PJI to control the depth of penetration and width of dispersal of the delivery [27]. The rapid liquid jet creates a hole in the skin at the initial explosion caused by the ignition powder, penetrates into the dermis, subcutaneous fat, or muscle depending on the amount of explosives, and then stops penetrating [27]. Then, during the second explosion induced by the smokeless powder, the liquid jet spreads radially and vertically in a concentric circle around the end of the formed hole, creating a strong shear stress [29]. Interestingly, shear stress has been demonstrated to facilitate the uptake and endocytosis of extracellular molecules [56,57]. Consistent with these reports, when luciferase expression plasmid was first injected intradermally with a needle syringe, the subsequent injection of saline at the same site by PJI greatly increased the luciferase activity compared with a needle syringe [29]. Furthermore, the induction of luciferase activity was inhibited by heparin, an inhibitor of the uptake of injected DNA [55], and by several endocytosis inhibitors [29], whereas treatment with chloroquine, which induces endosomal escape by rupture of endosomal vesicles via protonation of endosomes in an acidic environment [58], enhanced the induction of luciferase activity. These reports are in good agreement with the present findings in that shear stress induced by PJI facilitates protein uptake and endocytosis in XCR1^+^DCs, thereby inducing strong cross-presenting ability.

As naked mRNA induces strong lethal inflammatory responses, uridine nucleotides in the mRNA are replaced by pseudouridines to reduce the inflammatory responses, resulting in reduced antigenicity [59]. Therefore, the modified mRNA must be encapsulated in LNPs to reverse the reduced antigenicity [8]. The resulting mRNA-LNP complex is stable and is rapidly released from the endosomes after internalization to enhance antigen expression, acting as an adjuvant to stimulate potent innate immune responses. However, this strong immune activation also has adverse effects due to the reactogenicity associated with the wide distribution and circulation of mRNA-LNPs in the body after intramuscular injection, causing pro-inflammatory effects [9]. Therefore, alternative methods to enhance the antigenicity but reduce the systemic pro-inflammatory responses need to be developed. PJI is considered as one of the alternative candidates to LNP to enhance not only the delivery of the modified mRNA into cells but also their antigenicity. Notably, intradermal injection of naked mRNA using PJI without carriers such as LNP confined mRNA distribution to the injection site, preventing systemic pro-inflammatory responses [60]. This injection induced strong antigen-specific antibody production and T cell immune responses, comparable to but safer than the mRNA-LNP vaccines.

The advantage of using intradermal injection of PJI is that it provides a more effective and safer vaccination. Unlike chemical adjuvants, physical adjuvants such as PJI, which use physical energy to stress tissues, do not use chemical or biological substances and therefore do not persist in exposed tissues or cause lasting adverse effects [17,18,19]. In addition, the skin is an ideal site for vaccination because it contains a large number of immune cells, especially DCs [20,21]. However, the Phase I or Phase I/II clinical trial of PJI’s COVID-19 DNA vaccine suggested that further improvement of the efficacy of antibody production is still needed, although a cellular immune response was observed with no significant safety issues [61,62]. One of the reasons for the low humoral response may be due to the low dose of DNA used, and therefore further optimization of administration conditions is necessary. Regarding the durability of immune responses induced by PJI, it has been reported that intradermal injection of the plasmid expressing SARS-CoV-2 spike protein into rats three times every two weeks by PJI induced high titers of anti-spike protein antibodies that persisted for more than 12 weeks and neutralized SARS-CoV-2 infection in a mouse model [30]. Similarly, two intradermal injections of naked spike mRNA into mice 2 weeks apart by PJI induced spike-specific IgG levels that remained high for more than half a year, demonstrating durable humoral immune responses [60].

## 4. Materials and Methods

### 4.1. Mice

C57BL/6 female mice, 6~7 weeks old, were purchased from Sankyo Labo Service (Hamamatsu, Japan). All mice were maintained under pathogen-free conditions, and all animal experiments were approved by the President and the Institutional Animal Care and Use Committee of Tokyo Medical University and conducted in accordance with institutional, scientific community, and national guidelines for animal experimentation and the Animal Research: Reporting of In Vivo Experiments guidelines.

### 4.2. Cell Culture

E.G7-OVA cells (CRL-2113), a derivative of EL-4 thymoma cells transfected with OVA cDNA, were obtained from the American Type Culture Collection (Manassas, VA, USA). E.G7-OVA cells, lymph node cells, and splenocytes were cultured at 37 °C under 5% CO_2_/95% air in RPMI1640 medium containing 10% fetal bovine serum, l-glutamine (2 mM), penicillin (100 U/mL), streptomycin (100 μg/mL), and β-mercaptoethanol (50 μM).

### 4.3. Intradermal Injection

The PJI (called Actranza) was provided by the Daicel Corporation (Osaka, Japan), and 25 mg of ignition powder and 40 mg of smokeless powder were used for the mice [26,27,28,63,64]. After the abdominal skin of the mice was shaved, 20 μL of endotoxin-free OVA protein (1 mg/mL, EndoGrade^®^, Hyglos GmbH, Bernried, Germany) was injected intradermally into both the right and left flank areas twice at a 2-week interval (2 sites each, total 4 sites/mouse) using the PJI or a needle syringe (Micro-Fine Insulin Syringe 30G × 8 mm needle, BD Biosciences, Franklin Lakes, NJ, USA). OVA protein in PBS (1 mg/mL) was mixed with an equal volume of 2% aluminum hydroxide (AlumVax Hydroxide, OZ Biosciences, San Diego, CA, USA), and 20 μL of AlumVax-precipitated OVA protein (Alum/OVA) was injected subcutaneously into 2 sites on the dorsal skin.

### 4.4. Tetramer Staining Assay

Phycoerythrin-conjugated tetramers of MHC class I H-2K^b^ and its restricted peptide epitope of OVA (OVA_257–264_ peptide, SIINFEKL) and of MHC class II I-A^b^ and its restricted peptide epitope of OVA (OVA_323–339_ peptide, ISQAVHAAHAEINEAGR) were obtained from the MBL (Nagoya, Japan). After immunization, draining inguinal lymph node cells were stained with the MHC class I-OVA peptide tetramer and FITC-conjugated anti-CD8 (clone # KT15, MBL) or MHC class II-OVA peptide tetramer and FITC-conjugated anti-CD4 (clone # GK1.5, BioLegend, San Diego, CA, USA) according to the manufacturer’s protocol. The resulting cells were analyzed using a FACSCanto II flow cytometer (BD Biosciences) and the FlowJo software application (version 10: FloJo, Ashland, OR, USA).

### 4.5. In Vivo Killing Assay

C57BL/6 mouse splenocytes were incubated with and without MHC class I H-2K^b^-restricted OVA_257–264_ peptide (SIINFEKL, 1 μM) at 37 °C for 1 h and were washed with complete medium and PBS. The pulsed target splenocytes and unpulsed splenocytes were then incubated with, respectively, 5 μM and 0.5 μM carboxifluorescein diacetate succinimidyl ester (CFSE, Invitrogen, Carlsbad, CA, USA) for 10 min at 37 °C with gentle agitation [65]. After the cells were washed, equal numbers of peptide-pulsed CFSE^high^ cells and unpulsed CFSE^low^ cells (2 × 10^6^ cells each) were injected intravenously with the PJI or with a needle syringe into the mice to be vaccinated. Four hours later, spleens were harvested and analyzed using a FACSCanto II flow cytometer and the FlowJo software application.

### 4.6. Serum Antibody Titer

Blood was collected by cardiac puncture and the resulting serum was analyzed for concentrations of OVA-specific total IgG and IgG1 and IgG2a subclasses using anti-mouse IgG subtype/subclass ELISA kits (Chondrex, Woodinville, WA, USA; Bethyl Laboratories, Montgomery, TX, USA; Abcam, Cambridge, UK). OVA protein was coated onto an ELISA plate at 10 μg/mL in a carbonate buffer and incubated overnight at 4 °C. After blocking the plate with 5% BSA, serially diluted serum samples were added to the plate and incubated overnight at 4 °C, followed by incubation with horseradish peroxidase-conjugated anti-mouse total IgG, IgG1, and IgG2a. Color development was performed using a 3,3′,5,5′-tetramethylbenzidine substrate kit (BioLegend), and absorbance was detected at 450 nm in a microplate reader (iMark Microplate Reader, Bio-Rad, Hercules, CA, USA; GloMax^®^ Discover Microplate Reader, Promega, Madison, WI, USA).

### 4.7. In Vivo Tumor Growth

E.G7-OVA cells were harvested at the exponential growth phase and washed with PBS. Subsequently, 2 × 10^6^ cells/100 μL were injected subcutaneously into the right flank of C57BL/6 mice. Tumor size was measured daily using electronic calipers and was expressed as a volume (mm^3^) using the volume equation 0.5(*ab*^2^), where *a* is the long diameter and *b* is the short diameter. For the depletion of CD4^+^ T cells or CD8^+^ T cells, each mouse was injected i.p. with 0.5 mg rat anti-mouse CD8 (clone # 53-6.7), anti-CD4 (clone # GK1.5; all from American Type Culture Collection), or normal rat IgG (Sigma-Aldrich, St. Louis, MA, USA) as a control antibody, in 200 μL PBS, one day before tumor inoculation and once daily for the following 4 consecutive days as reported [66].

### 4.8. FACS Analysis of Skin and Lymph Node Cells

To detect DCs presenting the OVA peptide SIINFEKL in the MHC class I H-2K^b^ in the skin cells and lymph node cells, mice were injected intradermally with 20 μL Alexa Fluor 647-conjugated OVA (Thermo Fisher Scientific, Waltham, MA, USA) (1 mg/mL, 2 sites each) using PJI or a needle syringe. Twenty-four or forty-eight hours later, mouse abdominal skin tissues were excised, washed in PBS, minced and treated with 1 mg/mL dispase II (FUJIFILM Wako Pure Chemical, Tokyo, Japan) in RPMI1640 medium containing 5% fetal bovine serum at 4 °C overnight. The skin sample was further treated with 1 mg/mL collagenase (FUJIFILM Wako) at 37 °C for 2–3 h with shaking. After passing through a 70 mm strainer, the single cell suspension of skin cells and lymph node cells was then stained with Brilliant Violet 510 anti-mouse CD11b (clone # M1/70, BioLegend), PE-Cy7-conjugated anti-mouse CD86 (Clone # GL-1, BioLegend), PE anti-mouse XCR1 (clone # ZET, BioLegend), APC-Cy7-conjugated anti-mouse CD11c (clone # N418, BioLegend), and FITC-conjugated anti-mouse MHC class II (clone # M5/114.15.2, BioLegend). The resulting cells were analyzed on a FACSCanto II flow cytometer (BD Biosciences) using FlowJo software.

### 4.9. Western Blot Analysis

After intradermal injection of 20 μL OVA (1 mg/mL, 2 sites each) using PJI, the mouse abdominal skin tissue was excised, minced, and lyzed in a RIPA buffer (20 mM Tris-HCl pH 7.4, 150 mM NaCl, 1% Triton X-100, 1% sodium deoxycholate, and 0.1% SDS) containing protease inhibitor cocktail (Sigma-Aldrich) with sonication. After centrifugation, the protein concentration in the supernatant was determined using the BCA Protein Assay Kit (Takara, Shiga, Japan). The supernatant (5 μg/lane) was separated by SDS-PAGE under reducing conditions and transferred to polyvinylidene difluoride membrane (Millipore, Bedford, MA, USA). The membrane was then blocked, probed with anti-HMGB1 (Catalog # ab18256, Abcam, Cambridge, UK) or anti-actin (Sigma-Aldrich), followed by anti-mouse IgG conjugated to horseradish peroxidase, and visualized using the enhanced chemiluminescence detection system (Amersham Pharmacia Biotech, Piscataway, NJ, USA) according to the manufacturer’s instructions. Images were captured using an iBright FL1500 Imaging System (Thermo Fisher Scientific). The intensity of each band was quantified using ImageJ (version 1.53c; National Institutes of Health, Bethesda, MD, USA) [67].

### 4.10. Statistical Analysis

Data are expressed as the mean ± standard deviation (SD) for each group. Statistical analyses were performed in the GraphPad Prism software application (version 10: GraphPad Software, San Diego, CA, USA), using the unpaired two-tailed Student *t*-test for comparisons between two groups or a one- or two-way ANOVA with Tukey’s multiple comparison test for comparisons between three or more groups. *p* < 0.05 was considered to indicate a statistically significant difference.

## 5. Conclusions

In conclusion, intradermal injection of OVA protein alone without additional adjuvants using PJI induces potent CD8^+^ T cell-mediated antitumor immunity. The underlying mechanisms are strongly linked to the potent ability of PJI to induce shear stress, which enhances antigen uptake and endocytosis and also upregulates HMGB1, thereby facilitating cross-presentation in XCR1^+^ DCs. Thus, needle-free intradermal injections of DNA, mRNA or protein using PJI would be a promising strategy for more effective and safer vaccination against cancer and infections.

## Data Availability

Data is contained within the article.

## Supplementary Materials

The following supporting information can be downloaded at: https://www.mdpi.com/article/10.3390/ijms26094442/s1.

## Abbreviations

The following abbreviations are used in this manuscript:

ATP	adenosine triphosphate
Alum	aluminum hydroxide gel
CFSE	carboxifluorescein diacetate succinimidyl ester
DAMP	damage-associated molecular patterns
DC	dendritic cell
HMGB1	high mobility group box 1
HSP	heat shock protein
LNP	lipid nanoparticle
OVA	ovalbumin
PAMP	Pathogen-associated molecular pattern
PJI	pyro-drive jet injector;
RAGE	receptor for advanced glycation end products
TLR	toll-like receptor
