# Supplementary material for: Intradermal Injection of a Protein Alone Without Additional Adjuvants Using a Needle-Free Pyro-Drive Jet Injector Induces Potent CD8+ T Cell-Mediated Antitumor Immunity
Source: Int J Mol Sci. 2025 May 7;26(9):4442. doi: 10.3390/ijms26094442 (PMC12072794; doi:10.3390/ijms26094442)
Supplement: Supplementary file 1 [file ijms-26-04442-s001.zip › ijms-3615593-supplementary.pdf]

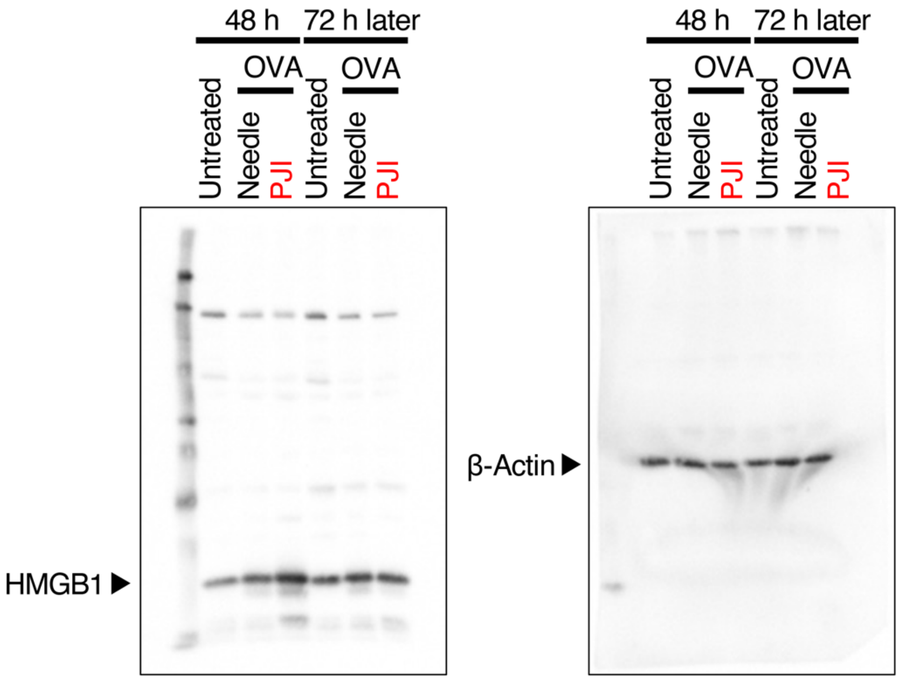

Supplementary Figure S1. Uncropped, untouched, full original images of the western blots from Figure 6B.
